# Fast Linde–Buzo–Gray (FLBG) Algorithm for Image Compression through Rescaling Using Bilinear Interpolation

**DOI:** 10.3390/jimaging10050124

**Published:** 2024-05-20

**Authors:** Muhammmad Bilal, Zahid Ullah, Omer Mujahid, Tama Fouzder

**Affiliations:** 1 Department of Information Engineering Technology, University of Technology, Nowshera 24170, Pakistan; mbilal@uotnowshera.edu.pk; 2 Department of Electrical and Computer Engineering, Pak-Austria Fachhochschule: Institute of Applied Sciences & Technology, Mang Haripur 22621, Pakistan; zahid.ullah@fecid.paf-iast.edu.pk; 3 Institut d’Informatica i Aplicacions, Universitat de Girona, 17003 Girona, Spain; 4 Department of Electrical and Electronic Engineering, Daffodil International University, Dhaka 1216, Bangladesh; tamafouzder.eee@diu.edu.bd

**Keywords:** computational time, codebook, firefly algorithm, bat algorithm, image compression, Linde–Buzo–Gray, peak signal to noise ratio, vector quantization

## Abstract

Vector quantization (VQ) is a block coding method that is famous for its high compression ratio and simple encoder and decoder implementation. Linde–Buzo–Gray (LBG) is a renowned technique for VQ that uses a clustering-based approach for finding the optimum codebook. Numerous algorithms, such as Particle Swarm Optimization (PSO), the Cuckoo search algorithm (CS), bat algorithm, and firefly algorithm (FA), are used for codebook design. These algorithms are primarily focused on improving the image quality in terms of the PSNR and SSIM but use exhaustive searching to find the optimum codebook, which causes the computational time to be very high. In our study, our algorithm enhances LBG by minimizing the computational complexity by reducing the total number of comparisons among the codebook and training vectors using a match function. The input image is taken as a training vector at the encoder side, which is initialized with the random selection of the vectors from the input image. Rescaling using bilinear interpolation through the nearest neighborhood method is performed to reduce the comparison of the codebook with the training vector. The compressed image is first downsized by the encoder, which is then upscaled at the decoder side during decompression. Based on the results, it is demonstrated that the proposed method reduces the computational complexity by 50.2% compared to LBG and above 97% compared to the other LBG-based algorithms. Moreover, a 20% reduction in the memory size is also obtained, with no significant loss in the image quality compared to the LBG algorithm.

## 1. Introduction

Images are significant representations of objects. They are utilized in many applications, such as digital cameras, satellite and medical imaging, or computer storage of pictures. Commonly, sampling, quantization, and encoding are performed on a 2D analog signal to generate a digital image that creates an abundant volume of data that is impractical for storing and transmission. To decrease the image’s size for storage and transmission, it is necessary to compress the image for practical use. The real-time transmission of images restricts the image compression techniques as they require fast buffering and low computational complexity [1]. Conversely, compressing images for storing in memory has no restrictions. It is due to this that the algorithms are executed in non-real time, where there is no need for buffers for the communication channel [2]. Image compression can be categorized into two main types: one is lossless, which contains no loss of image quality and is used for applications where no loss is tolerable, such as medical imaging, scientific research, and satellite imaging, while the other is lossy, which bears the loss in quality and is suitable for applications where losses are acceptable, such as video streaming, web publishing, and social media, etc. [3]. The goal of compressing images is to reduce the storage requirement of the images.

A lossless image compression algorithm formulated by Garcia et al. [4] is a renowned technique that uses Huffman coding [5] (hierarchical encoding) to assign variable coding procedures in which more bits are assigned to less frequently occurring data and fewer bits to more frequently occurring data. The Huffman coding algorithm is adopted in many standards, including the JPEG (Joint Photographic Experts Group) [6]. However, it is efficient only if the probabilities of the data are provided and the data encoding is performed integrally. As the histograms vary from image to image, it is thus not certain whether the Huffman algorithm performs optimally. Another well-known technique of lossless image compression is arithmetic coding [7], which contains variations in coding just like the Huffman algorithm. It is based on reducing the redundant codes present in the image data. It performs efficiently if the probability of the occurrence of each symbol is identified. Arithmetic coding works on the principle of generating an interval for every symbol, which is calculated through cumulative probability. It assigns intervals to symbols from high to low and rescales the rest of the intervals until all the symbols are rescaled. It is an error-sensitive technique; a one-bit error will corrupt the entire image [8]. Lossless prediction coding is commonly applied in image coding to eliminate inter-pixel redundancy [9], ensuring accurate reconstruction without the loss of data. It predicts the current value of the pixels from the neighboring pixels and creates new values from the predicted value and original pixel values.

**Figure 1:**
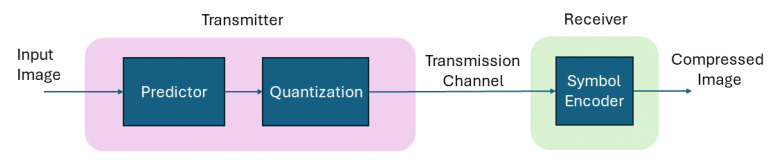
High-level block diagram of predictive coding.

On the other hand, lossy image compression techniques outperform lossless techniques in terms of compression [10]. There are wide applications where the loss in the image is tolerable and lossy image compression is preferred due to its tendency to lower the bit rate as desired by the application. Various lossy schemes are proposed, including predictive coding [11], which performs predictions using neighboring pixels. It performs quantization to an error message obtained through the predictive and actual values of an image as shown in Figure 1.

If the prediction is accurate, the encoding will produce high compression. Adding many pixels in the prediction process will directly affect the computational cost, and it has been noticed that, after attaining three and above previous pixels, no substantial improvement is observed in the image compression, as observed in algorithms such as the JPEG (Joint Photographic Experts Group). Transform coding is extensively employed for image compression, involving the conversion from one domain to another, resulting in densely packed coefficients [12]. Some coefficients exhibit high energy, while others have low energy. Transform coding techniques aim to efficiently pack the information using a limited number of coefficients, with a quantization process used to discard coefficients containing minimal information. The Karhunen–Loeve Transform (KLT) [13] is another technique used for compression that uses vectors with low-subspace dimensions. It uses correlation vectors, which are constructed using the original image. These correlation vectors are used to calculate the orthogonal vectors. These vectors are used as a linear combination to represent the original image. The KLT is not an ideal coding scheme due to image interdependences and computation. Another important lossy technique is the discrete cosine transform, which is orthogonal and transforms the image to the frequency domain. Such a representation is relatively compact and has the advantage of better results in discontinuous data at the end blocks compared to the Discrete Fourier Transform [14]. The DCT is adopted in many standards, including the Joint Photographers Expert Group (JPEG). The JPEG was initiated in 1987 and became an international standard in 1992 by the International Standards Organization (ISO) [15]. The standard contains four modes, including hierarchical, progressive, baseline, and lossless. The baseline mode is the default mode, and it is a common standard adopted worldwide. It has a two-step model in which the first DCT is applied and a quantization process is performed to remove the psycho-visual redundancy. In the second step, the entropy encoders are used to to remove the coding redundancy.

JPEG 2000 was developed after its first release, and it was a joint venture of the ISO and the International Telecommunication Union (ITU). It has an advantage over the JPEG due to its high compression ratio and better functionalities. It differs from the JPEG in terms of the transform coefficients and provides additional functions regarding progressive resolution transmission, better quality, error calculation, and information on location.

One of the renowned image compression techniques is vector quantization, which is recognized for its effectiveness in achieving high compression ratios with minimal distortion at specified bit rates [16]. It offers many features, including high compression with a higher block size and low compression with a smaller block size. The alteration in distortion can be tailored to specific applications by modifying the size of the block. Moreover, it provides a rapid decompression method utilizing codebooks and indexes, suitable for implementing in web and multimedia applications to decompress the images multiple times. Generating the optimal codebook is a pivotal aspect of vector quantization, and it can be refined through various optimization techniques, including the implementation of genetic algorithms [17], ant colony optimization [18], and other various optimization techniques. Linde–Buzo–Gray [19] developed an algorithm that recursively produces an optimal codebook of size “N” from a random selection of codebooks. It divides the original vectors into N number of clusters and endeavors to refine the codebook until the distortion is within the acceptable threshold.

Several optimization algorithms are applied on LBG for codebook generation and its optimization; however, these algorithms improved the quality of the reconstructed image but suffered greatly in terms of computational time [20]. Hence, in the proposed research, a fast-LBG-based codebook generation is proposed, which improves the computational time and storage requirements of LBG and LBG-based algorithms.

## 2. Recent Algorithms for Codebook Generation

**Figure 2:**
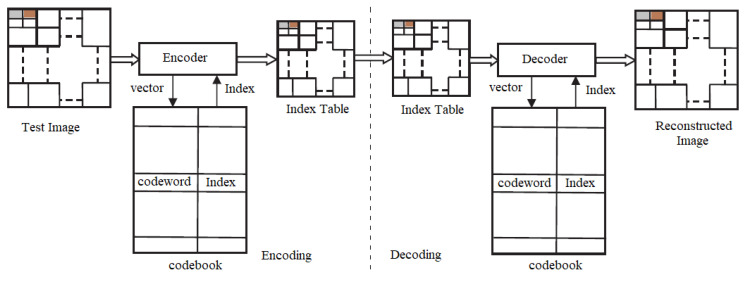
Block diagram of VQ encoder and decoder.

Vector quantization is a block coding method using encoding to find the final codebook. The codebook contains the codeword and indexes, which are transmitted to the receiver. The decoder uses indexes and codewords to reconstruct the image. The encoder and decoder of the VQ are shown in Figure 2.

The test image is configured to match a training vector of dimensions (N×N), which is then condensed into a smaller block of size $N_{b}$ (n×n). The blocks are represented as $X_{i}$, where (*i* = 1, 2, 3, 4……..$N_{b}$). Specific vectors from the $N_{b}$ blocks are chosen as the codewords. These vectors are selected based on minimum “D” between codeword and training vector, and they are denoted as $C_{j}$ (*i* = 1, 2, 3…….$N_{c}$). In this context, $N_{c}$ denotes the overall number of codewords within the codebook. The index of the codebook represents the location of each codeword in the codebook, which is updated during each iteration after the “D” is calculated. After the finalization of indexes and codewords, they are combined and transmitted as a codebook to the receiver. The decoder after receiving the codebook uses index and codewords for reconstructing the original image. The distance of the codebook with the training vector is calculated as

(1) $$Euclidean\;Distance=D=1/N_{c}\sum_{j=1}^{Nc}\sum_{i=1}^{Nb}V_{ij}*||X_{i}-C_{j}{\left|\right|}^{2}$$

Under condition

(2) $$D=\sum_{j=1}^{N_{c}}V_{ij}=1,\;\mathrm{for}\;\mathrm{all}\;i\in \left\{1,2,3,\cdots ,N_{b}\right\}$$

(3) $$V_{ij}=\left\{\begin{array}{cc} 1, & \mathrm{if}\;X_{i}\;\mathrm{belongs}\;\mathrm{to}\;\mathrm{the}\;j\mathrm{th}\;\mathrm{cluster}\\ 0, & \mathrm{otherwise} \end{array}\right.$$

For VQ, it is important to satisfy two conditions,

(1) A partition denoted by $R_{j}$ for all $j=1,2,3,4,\ldots ,N_{c}$ will satisfy the given criteria.

(4) $$R_{j}⊃\{x\epsilon X;d\left(c,C_{j}\right)<d\left(x,C_{k}\right),if\;\forall k\neq j$$

(2) The $C_{j}$ defines a centroid $R_{j}$ where

(5) $$C_{j}=1/N_{j}\sum_{i=1}^{Nj}x_{i},\forall x_{i}\epsilon R_{j}$$

Here, $N_{j}$ represents total vectors that belong to $R_{j}$.

### 2.1. Vector Quantization Using LBG

The first algorithm to apply the VQ technique, as described by Linde et al. (1980), is known as the Linde–Buzo–Gray (LBG) algorithm [21]. Algorithm 1 illustrates the steps of the LBG algorithm. This method employs a k-means clustering approach, utilizing a proximity function to identify the optimal local solution. This function makes an effort to prevent distortion from becoming worse between iterations. Due to its ineffective randomly initialized codebook, this technique becomes trapped in local optima and is unable to find the optimal solution.
**Algorithm 1:** LBG AlgorithmRk→CBInitialize *X* = ($x_{1}$, $x_{2}$…$x_{k}$) as initial training vectors. The Euclidean Distance (*D*) among the two vector is D(x,y).Step 1: Initial codebook $CB_{0}$, which is generated randomly.Step 2: Initialize *i* = 0.Step 3: Execute the given steps for each training vector. Calculate the distances among the codewords in CBi and training vector as *D*(*X*; *C*) = (x*_t_* − c*_t_*).Find the closest codeword in CBi.Step 4: Divide the codebook in clusters of N number of blocks.Step 5: Calculate the centroid of each block for obtaining the new codebook CBi + 1.Step 6: Calculate the average distortion of CBi + 1. If no improvement in last iteration, the codebook is finalized and execution stops. Otherwise, *i* = *i* + 1, and go to Step 3.

### 2.2. Particle Swarm Optimization Vector Quantization Algorithm

Hsuan and Ching proposed Particle Swarm Optimization (PSO) to find the optimum codebook for VQ [3]. The codebook employs swarm intelligence to adjust the codewords based on the natural behavior principles observed in schools of fish, as depicted in Algorithm 2. This approach offers a global codebook, in case the particle update velocity is kept higher, but requires many iterations to find the global best solution.
**Algorithm 2:** PSO-LBG AlgorithmStep 1: Implement the LBG algorithm to discover the codebook and designate as the global best (gbest) codebook.Step 2: Randomly generate additional codebooks.Step 3: Compute the fitness values for each codebook.
(6)$$Fitness\left(C\right)=\frac{1}{DistortionD(C)}$$
(7)$\frac{1}{D(C)}=\frac{N_{b}}{\sum_{J=1}^{Nc}\sum_{i=1}^{Nb}u_{ij}*||X_{i}-C_{j}{\left|\right|}^{2}}$Step 4: Upon observing an enhancement in the fitness of the codebook compared to the previous fitness (pbest), assign the new fitness value as pbest.Step 5: Identify the codebook with the highest fitness value; if the fitness surpasses that of gbest, update gbest with the new value.Step 6: Update velocities and elements to transition to a new position.
(8)$$V_{ik}^{n+1}=V_{ik}^{n}+C_{1}r_{1}^{n}\left(pbest_{ik}^{n}-X_{ik}^{n}\right)+C_{2}r_{2}^{n}\left(gbest_{k}^{n}-X_{ik}^{n}\right)$$
(9)$$X_{ik}^{n+1}=X_{ik}^{n}+V_{ik}^{n+1}$$The variable K denotes the total number of solutions, where “*i*” denotes the position of a particle, and $r_{1}$ and $r_{2}$ represent random numbers, while $C_{1}$ and $C_{2}$ signify the rates of social and cognitive influences, respectively.Step 7: Until max iteration or stopping criteria are met, repeat Steps 3–7.

### 2.3. Quantum-Inspired Particle Swarm Optimization Vector Quantization Algorithm

By the procedure outlined in Algorithm 3, Wang et al. implemented the Quantum Swarm Evolutionary Algorithm (QPSO) [22], whereby the local points are estimated as $P_{i}$ utilizing Equation (10) derived from the local best (pbest) and global best (gbest) codebooks. The adjustment of particle positions is facilitated by manipulating parameters u and z. It is noted that refining these parameters to improve PSNR entails substantial computational resources, surpassing those demanded by PSO and LBG algorithms.

(10) $$P_{i}=r_{1}pbest_{i}+r_{2}gbest_{i}/r_{1}+r_{2}$$

### 2.4. Firefly Vector Quantization Algorithm

The firefly algorithm for codebook design was introduced by MH Horng [23]. This algorithm, inspired by the flashing behavior of fireflies, incorporates brightness into its objective function. It operates by generating multiple codebooks, analogous to fireflies, with the goal of transitioning from lower to higher intensities or brightness values. However, if there is a lack of brighter fireflies within the search space, the algorithm’s performance in terms of the PSNR may deteriorate. The FA-VQ algorithm is depicted as Algorithm 4 in the present study.
**Algorithm 3:** QPSO-LBG AlgorithmStep 1: Initialization of the LBG algorithm involves assigning the global best codebook (gbest) and initializing the remaining codebooks and velocities randomly.Step 2: The fitness of each codebook is computed.Step 3: If the newly computed fitness surpasses the previous best fitness (pbest), the new fitness value replaces pbest.Step 4: The largest fitness value among all particles is taken, and, if an improvement is detected in gbest, it is updated with the new value.Step 5: Random values, r1, r2, and *u* are chosen within the range of 0 to 1, and the local point Pi is calculated using Equations (8) and (9).Step 6: The elements of the codebook Xi are updated according to Equations (11)–(13).
(11)$$L_{i}=z\left|X_{i}-p_{i}\right|$$
(12)$$if\;u>0.5X_{i}\left(t+1\right)=p_{i}-L_{i}*ln\left(1/u\right)$$
(13)$else\;X_{i}\left(t+1\right)=p_{i}+L_{i}*ln\left(1/u\right)$In this context, the constant ‘*z*’ is maintained such that it satisfies the condition *z* < 1/ln√2, where ‘*t*’ represents the iterations.Step 7: Steps (3) to (7) are iterated until reaching the maximum allowable number of iterations.

**Algorithm 4:** FA-LBG Algorithm
Step 1: Implement the LBG algorithm and designate its output as the brighter firefly (codebook).
Step 2: Initialize the parameters alpha (α), beta (β), and gamma coefficients (λ).
Step 3: Randomly initialize codebooks; select maximum iteration count *j*.
Step 4: Start count m=1.
Step 5: Evaluate the fitness of all codebooks using Equation (6). Choose a codebook randomly based on its fitness value and commence moving codebooks toward the brighter fireflies using Equations (14)–(17).
(14)$$Euclidean\;distance\;r_{ij}=\left|\right|X_{i}-X_{j}\left|\right|$$
(15)$$\left|\right|X_{i}-X_{j}\left|\right|=\sqrt{\sum_{k=1}^{N_{c}}\sum_{h=1}^{L}{\left(X_{ik}^{h}-X_{jk}^{h}\right)}^{2}}$$
(16)$$\beta =\beta_{o}e^{-\gamma ij}$$
where 0 < *u* < 1 and *k* = (1, 2, 3,…..$N_{c}$).
Step 6: If brighter fireflies cannot be located, begin moving randomly within the search space in pursuit of brighter ones using
(17)$$X_{jk}^{h}=\left(1-\beta \right)X_{ik}^{h}+\beta X_{jk}^{h}+u_{jk}^{h}$$
Step 7: If (*m* = *j*), execution stops.
Step 8: Increment *m* = *m* + 1.
Step 9: Jump to step 5.


### 2.5. BA Vector Quantization Algorithm

Karri et al introduced a bat algorithm (BA) for vector quantization, inspired by the mating behavior of bats [24]. In this algorithm, the codebook is considered as a bat, estimating the global codebook through three key parameters: loudness, frequency, and pulse rate. Compared to other LBG-based algorithm techniques, it achieves notably high PSNR. Nonetheless, it requires the calculation of an extra parameter, leading to a notable rise in computation duration when contrasted with PSO, QPSO, and LBG algorithms. Algorithm 5 introduces the BAT algorithm.
**Algorithm 5:** BA-LBG AlgorithmStep 1: Begin by allocating N codebooks, represented as bats, and defining parameters ‘A’ as (loudness), ‘V’ as (velocity), ‘R’ as (pulse rate), ‘Qmin’ as (minimum frequency), and ‘Qmax’ as (maximum frequency).Step 2: Implement the LBG algorithm to establish the initial codebook. Randomly select the remaining codebooks, denoted as $X_{i}$ (where *i* = 1, 2, 3,...*N*− 1).Step 3: Set the iteration counter m to 1 and define the maximum count as j.Step 4: Evaluate all codebooks’ fitness values using Equation (6). Identify $X_{bst}$ as the best-performing codebook.Step 5: Update the positions of the codebooks by adjusting their frequency and velocity according to Equations (18) through (20).
(18)$$Q_{1}\left(t+1\right)=Q_{\mathrm{maximum}}\left(t\right)+\Delta Q\left(t\right)$$
where $\Delta Q\left(t\right)=(Q_{\mathrm{minimum}}\left(t\right)-Q_{\mathrm{maximum}}\left(t\right))\cdot \left(R\right)$
(19)$$V_{1}\left(t+1\right)=V_{i}\left(t\right)+\Delta V\left(t\right)$$
where $\Delta V\left(t\right)=\left(X_{i}\left(t\right)-X_{\mathrm{best}}\left(t\right)\right)\cdot Q_{i}\left(t+1\right)$
(20)$X_{1}\left(t+1\right)=X_{i}\left(t\right)+V_{i}\left(t\right)$Step 6: Select the size of the step between 0 and 1 for the random walk (W).Step 7: If the step size exceeds the pulse rate (*R*), the codebook is shifted using Equation (21).
(21)$$X_{1}\left(t+1\right)=X_{best}\left(t\right)+W*R$$Step 8: Produce a randomized value, and, if its magnitude is below the threshold of loudness, incorporate it into the codebook.Step 9: Perform sorting the codebooks with respect to fitness value $X_{best}$.Step 10: If the condition (*m* = *j*) is satisfied, the execution halts. Otherwise, the value of m is incremented by 1.

## 3. Proposed Fast-LBG Algorithm

**Figure 3:**
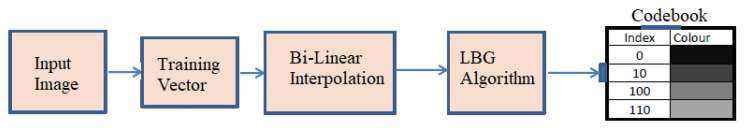
Block diagram of the proposed fast-LBG algorithm.

The conventional LBG algorithm faces inefficiencies because of the random initialization of the initial codebook, often leading to entrapment in local optima. While PSO and QPSO methods generate efficient codebooks, higher-velocity particles may suffer from instability. Constructing a codebook for HBMO [25] demands numerous tuning parameters. Additionally, the FA algorithm’s convergence is compromised when there are no brighter fireflies [26] in the search space. Failure to meet the convergence conditions in the CS algorithm [20] necessitates numerous iterations. To address these challenges, a novel approach is proposed, modifying the LBG algorithm by incorporating rescaling via bilinear interpolation to reduce the computational time while maintaining a PSNR and SSIM near the LBG algorithm. The proposed method’s block diagram is depicted in Figure 3.

### Bilinear Interpolation for Codebook Rescaling

Bilinear interpolation allows for the estimation of a function’s value at any location within a rectangle given that its value is known at each of the rectangle’s four corners. This technique is particularly relevant in our methodology, where resizing the image is essential for reducing computational complexity. In our proposed method, bilinear interpolation is employed using the nearest neighborhood method to rescale the image and reduce the comparison between the codebook and the training vector. Bilinear interpolation estimates the value of an unknown function at a specific location (*x*, *y*) based on the known values at four surrounding sites. The encoder uses the input image as a training vector, which is initialized with a random selection of vectors from the image. Assume we want to find the value of the unknown function *f* at a specific location (x,y). The values of *f* at the four sites Q11=(x1,y1), Q12=(x1,y2), Q21=(x2,y1), and Q22=(x2,y2) are assumed to be known. First, a bilinear interpolation is performed in the *x* direction using the following equation:

(22) $$f\left(x,y_{1}\right)=\frac{x_{2}-x}{x_{2}-x_{1}}f\left(Q_{11}\right)+\frac{x-x_{1}}{x_{2}-x_{1}}f\left(Q_{21}\right)$$

To acquire the necessary estimate, interpolation is performed in the *y*-direction using the following equation:

(23) $$f\left(x_{1},y\right)=\frac{y_{2}-y}{y_{2}-y_{1}}f\left(Q_{12}\right)+\frac{y-y_{1}}{y_{2}-y_{1}}f\left(Q_{22}\right)$$

Through interpolation, we reduce the size of the image by 1/4 of the original image size. This size reduction reduces the total number of comparisons between the training vector and the codebook. The decoder performs upscaling in response to the encoder’s downscaling. The upscaling is performed by a factor of 4, and the index at the decoder is used to replicate the image pixels at the receiving end to match the size of the compressed image with the original image. Based on the findings, it can be concluded that the proposed methods reduce computing complexity. The proposed algorithm is shown in Algorithm 6.
**Algorithm 6:** Proposed FLBG AlgorithmStep 1: Resize the image using bilinear interpolation using Equations (25) and (26).Step 2: Find the Euclidean Distance “*D*” between the two vectors as *D*(*x*,*y*).Step 3: Initial codebook $CB_{0}$, which is generated randomly.Step 4: *i* = 0.Step 5: Execute the given steps for each training vector. Calculate the distances among the codewords in CBi and the training vector as *D*(*X*; *C*) = (x*_t_* − c*_t_*).Find the closest codeword in CBi.Step 6: Divide the codebook into clusters of N number of blocks.Step 7: Calculate the centroid of each block for obtaining the new codebook CBi + 1.Step 8: Calculate the average distortion of CBi + 1. If no improvement in last iteration, the codebook is finalized and execution stops, or else *i* = *i* + 1, and go to Step 4.

## 4. Results and Discussion

**Figure 4:**
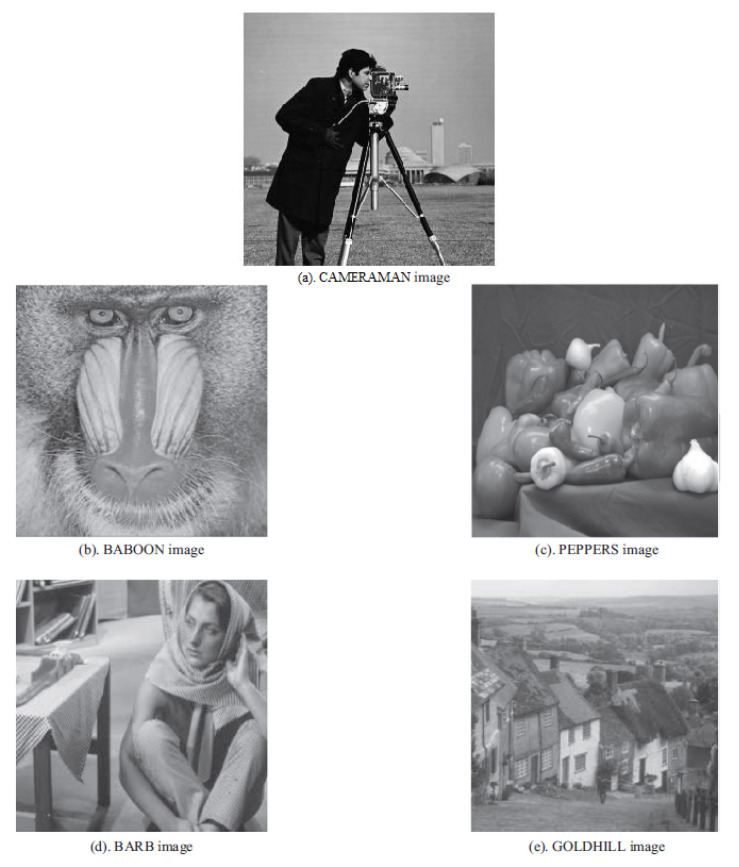
(**a**–**e**) The images utilized for analytical purposes underwent compression during the experimentation.

The evaluation of the codebook involved conducting tests on grayscale images. For the comparative study, five distinct test photographs shown in Figure 4 were utilized: ‘Cameraman.png’, ‘Baboon.png’, ‘peppers.png’, ‘Barb.png’, and ‘Goldhill.png’. The simulations were performed on a 32-bit Windows 11 Pro system using an Intel^®^ Core™ i5-3210M Processor running at 2.54 GHz with a 3M Cache 4 GB double data rate 3 RAM. MATLAB version R2019A was utilized for compiling the codes. All the tests were conducted on 512×512 grayscale images, as depicted Figure 4.

Initially, the test image undergoes partitioning into non-overlapping blocks measuring 4×4 pixels each for compression purposes. These blocks are then considered as 16,384-dimensional training vectors, with the dimension of each input vector set to 16. The comparison metrics utilized include data rate per pixel (BPP), Mean Square Error (MSE), and peak signal-to-noise ratio (PSNR), calculated using Equations (24), (25), and (26), respectively.

(24) $$bpp=\frac{Log_{2}N_{c}}{k}$$

*k* represents size of the block, while $N_{c}$ indicates the size of codebook. The bit rate normalized by the number of pixels serves as a metric for evaluating the magnitude of compression in an image.

(25) $$MSE=\frac{1}{M\times N}\sum_{i=1}^{M}\sum_{j=1}^{N}{\left(f\left(i,j\right)-\hat{f}\left(i,j\right)\right)}^{2}$$

M×N denotes the overall pixel count, where *I* and *J* signify the *x* and *y* coordinates of pixel values. The test image is referenced as f(I,J), while the compressed image is denoted as *f*−(*I*, *J*).

(26) $$PSNR=10\;log_{10}{\left(\frac{255}{MSE}\right)}^{2}\;\left(db\right)$$

PSNR measurements are employed for assessing the quality of the decompressed image. Five test images are examined, each standardized to a size of M×N (512×512) pixels while employing varied codebook sizes (8, 16, 32, 64, 128, 256, 512, and 1024). The maximum average PSNR values are utilized for determining the parameters in the proposed method for simulating the test image, which is executed four times. Comparative Table 1, Table 2, Table 3, Table 4 and Table 5 depict the PSNR evaluation of the test images utilizing FLBG in comparison to the existing algorithms. The analysis of the average variation in peak signal-to-noise ratio concerning the bit rate indicates that the proposed algorithm achieves a PSNR level comparable to that of LBG.

**Figure 5:**
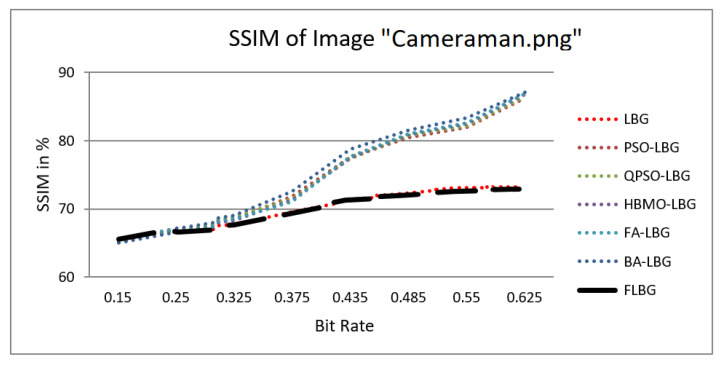
Similarity index measure for Cameraman image.

**Figure 6:**
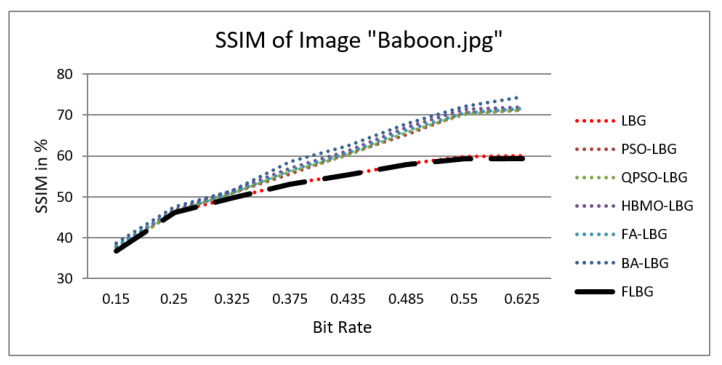
Similarity index measure for Baboon image.

**Figure 7:**
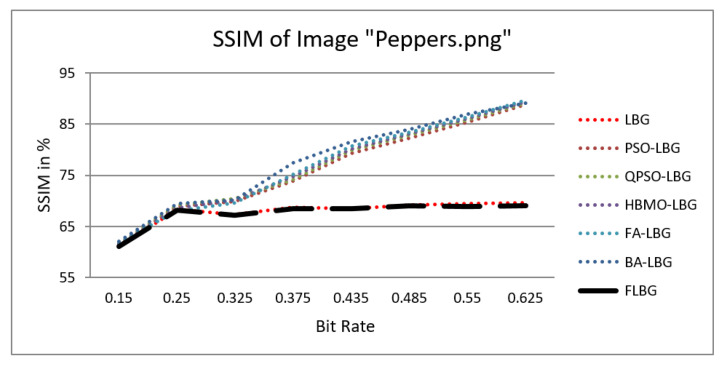
Similarity index measure for Peppers image.

**Figure 8:**
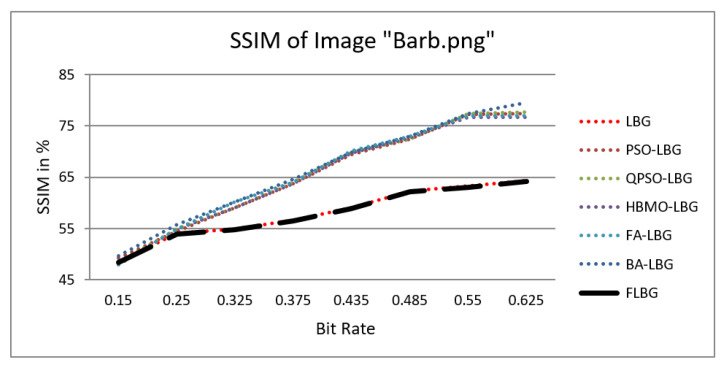
Similarity index measure for Barb image.

**Figure 9:**
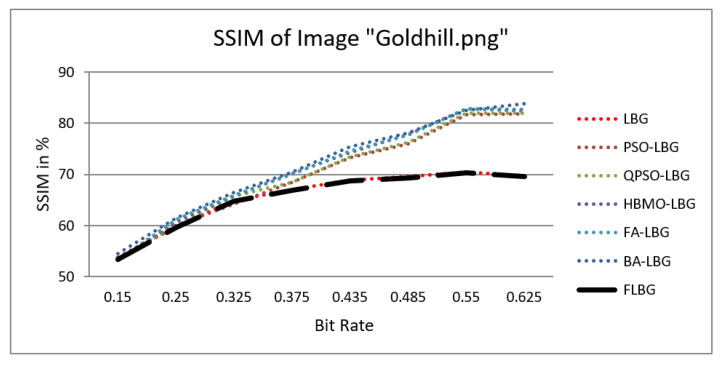
Similarity index measure for Goldhill image.

Although PSNR is a valuable metric for comparing image quality, it may not fully correlate with human visual perception, especially in distinguishing structural details. To overcome this limitation and facilitate structural comparison, we computed the Structural Similarity Index Measure (SSIM) metrics [27]. These metrics evaluate luminance, contrast, and structure among the test images and the compressed images. The SSIM score is determined using Equation (27).

(27) $$SSIM\left(X,Y\right)={\left[L\left(X,Y\right)\right]}^{\alpha }.{\left[C\left(X,Y\right)\right]}^{\beta }.{\left[S\left(X,Y\right)\right]}^{\gamma }$$

where *S*, *C*, and *L* denote structure, contrast, and luminance, respectively, while alpha, beta, and gamma signify the relative significance of these parameters. For the sake of ease, it was presumed that alpha = beta = gamma = 1. The SSIM values were observed to vary between 0 and 1, where 0 indicates no similarity and 1 signifies a high degree of resemblance between the two images. As shown in Figure 5, Figure 6, Figure 7, Figure 8 and Figure 9, using FLBG, the SSIM scores (expressed as percentages) of five test images are compared to those obtained from existing algorithms.

**Figure 10:**
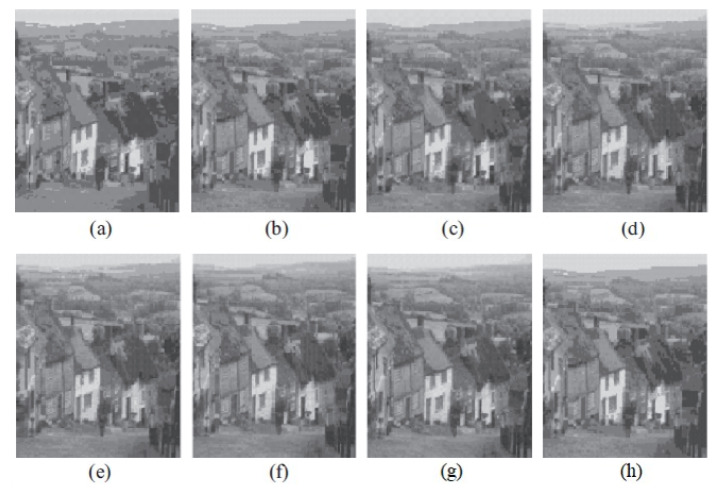
The image Goldhill reconstructed employing six distinct algorithms: (**a**) Linde–Buzo–Gray. (**b**) Linde–Buzo–Gray Particle Swarm Optimization. (**c**) Linde–Buzo–Gray Quantum Particle Swarm Optimization. (**d**) Linde–Buzo–Gray Honey Bee Mating Optimization. (**e**) Linde–Buzo–Gray firefly algorithm. (**f**) Linde–Buzo–Gray bat algorithm. (**g**) Linde–Buzo–Gray Cuckoo Search Optimization. (**h**) Fast Linde–Buzo–Gray.

**Figure 11:**
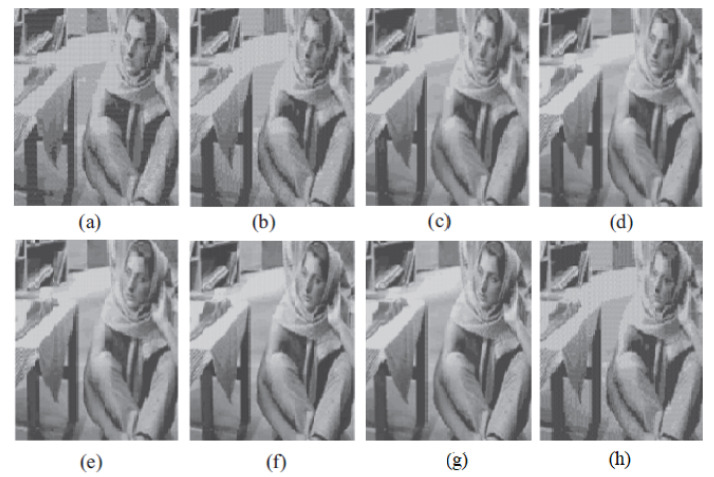
The image of Barb reconstructed employing six distinct algorithms: (**a**) Linde–Buzo–Gray. (**b**) Linde–Buzo–Gray Particle Swarm Optimization. (**c**) Linde–Buzo–Gray Quantum Particle Swarm Optimization. (**d**) Linde–Buzo–Gray Honey Bee Mating Optimization. (**e**) Linde–Buzo–Gray firefly algorithm. (**f**) Linde–Buzo–Gray bat algorithm. (**g**) Linde–Buzo–Gray Cuckoo Search Optimization. (**h**) Fast Linde–Buzo–Gray.

**Figure 12:**
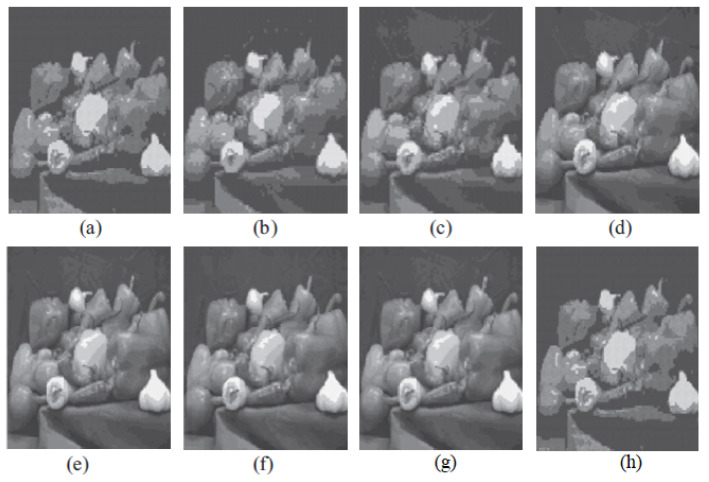
The image of Peppers reconstructed utilizing six distinct algorithms: (**a**) Linde–Buzo–Gray. (**b**) Linde–Buzo–Gray Particle Swarm Optimization. (**c**) Linde–Buzo–Gray Quantum Particle Swarm Optimization. (**d**) Linde–Buzo–Gray Honey Bee Mating Optimization. (**e**) Linde–Buzo–Gray firefly algoorithm. (**f**) Linde–Buzo–Gray bat algorithm. (**g**) Linde–Buzo–Gray Cuckoo Search Optimization. (**h**) Fast Linde–Buzo–Gray.

The graph indicates that the proposed algorithm achieves an SSIM percentage that is approximately equal to that of the LBG algorithm. Figure 10, Figure 11 and Figure 12 examine and contrast three reconstructed test images utilizing FLBG- and LBG-derived algorithms, employing a codebook capacity of 64 and a block size of 16. It is observed that the image quality of the reconstructed images depicted in Figure 10, Figure 11 and Figure 12 is comparable to that of LBG. Simulations were conducted by varying the codebook sizes. Increasing the codebook size enhances the image quality but also increases the total number of comparisons among codewords and training vectors, resulting in longer computation times and lower compression ratios. Nevertheless, a notable reduction in computation time was achieved during testing. Table 6 presents the average processing times for various test images measured using the FLBG and comparable algorithms. Each test image was run six times to compute the average processing time for accurate evaluation. The results demonstrated a significant efficiency advantage of FLBG over traditional LBG and other LBG-based algorithms. Specifically, FLBG achieved a 47.7% reduction in processing time compared to LBG, showcasing a substantial improvement. Furthermore, FLBG outperformed other LBG-based algorithms by more than 97%, indicating its superior performance.

It can be observed from these tables that FLBG has less computational time and reduced image size compared to the LBG-based algorithms, such as HBMO-LBG, FA-LBG, BA-LBG, PSO-LBG, QPSO-LBG, and CS-LBG.

It is important to mention here that this work specifically focuses on an LBG compression method that is a VQ-based technique. We acknowledge that its focus may seem narrow in comparison to the widely used transform-based methods like the JPEG, JPEG 2000, and WebP. However, we would like to emphasize that our intention was not to directly compete with these established algorithms in terms of computational time or file size reduction. Instead, our aim was to enhance the computational speed of the VQ-based LBG compression method.

While it is true that comparing the resultant image sizes and execution times of various algorithms is crucial for selecting the most suitable compression method, we believe that a qualitative comparison is equally important, especially when considering different algorithmic approaches. Transform-based methods excel in many scenarios, but there are specific cases where VQ methods offer unique advantages, such as preserving perceptual quality, exploiting correlated data, and facilitating fixed-rate compression.

In this work, we sought to highlight the importance of VQ methods in certain application domains where these advantages are critical. Although our enhancements may not directly improve the execution time or file size reduction compared to the state-of-the-art transform-based methods, they contribute to the broader discussion on the relevance and necessity of VQ techniques.

## 5. Conclusions

A fast-LBG algorithm is proposed for compressing images, wherein the codebook generation is enhanced by pre-scaling the image prior to applying the LBG algorithm. This pre-scaling optimizes the process by exploiting the inherent high correlation among the pixels, thus reducing inter-pixel redundancies. The reduction in the training vector size before the LBG algorithm application significantly cuts the processing time by minimizing the number of required comparisons among the training vector and codebook codewords. Consequently, the resultant image size is contingent upon the chosen rescaling factor, enabling increased compression potential at the expense of image quality.

Based on the simulation outcomes, it is evident that the proposed algorithm excels in terms of computational efficiency, advocating for its adoption in LBG and LBG-based algorithms to mitigate the computational complexity and diminish compressed image dimensions.

The potential avenues for future research encompass exploring the efficacy of the algorithm on polychrome, monochrome, and three-dimensional images. Moreover, employing advanced scaling mechanisms such as edge-directed interpolation or Sinc and Lanczos resampling could further enhance the results.

## Figures and Tables

**Table 1:** Image ‘Cameraman’ PSNR vs. bitrate comparison.

Bpp	PSNR in Decibels						
	**LBG**	**PSO**	**QPSO**	**HBMO**	**FA**	**BA**	**FLBG**
0.15	25.2	25.4	25.2	25.4	25.3	25.5	25.3
0.25	26.4	26.3	26.4	26.5	26.4	26.5	26.3
0.325	26.4	26.5	26.5	26.6	26.4	26.5	26.4
0.375	26.2	26.7	27.2	26.9	26.8	27.35	26.2
0.435	26.3	28.6	28.6	28.5	28.7	29.2	26.5
0.485	26.5	29.8	29.4	29.5	29.7	29.9	26.6
0.55	26.7	30.2	30.2	30.2	30.1	30.5	26.8
0.625	26.7	31.4	31.5	31.6	31.6	31.8	26.8

**Table 2:** Image ‘Baboon’ PSNR vs. bitrate comparison.

Bpp	PSNR in Decibels						
	**LBG**	**PSO**	**QPSO**	**HBMO**	**FA**	**BA**	**FLBG**
0.15	18.2	18.3	18.1	18.7	19.1	19.1	18.1
0.25	19.6	19.6	19.7	19.8	19.6	20.1	19.3
0.325	19.5	20.2	20.2	20.1	20.2	20.2	19.4
0.375	19.6	20.5	20.7	21.2	20.8	21.6	19.2
0.435	19.7	21.5	21.4	21.8	21.6	22.2	19.1
0.485	19.7	22.1	22.3	22.7	22.5	23	19.3
0.55	19.6	23.1	23.2	23.4	23.1	23.6	19.4
0.625	19.7	23.4	23.4	23.6	23.5	24.4	19.4

**Table 3:** Image ‘Peppers’ PSNR vs. bitrate comparison.

Bpp	PSNR in Decibels						
	**LBG**	**PSO**	**QPSO**	**HBMO**	**FA**	**BA**	**FLBG**
0.15	24.2	24.3	24.4	24.4	24.4	24.6	24.1
0.25	25.1	25.3	25.4	25.2	25.2	25.5	24.8
0.325	25.2	26.2	26.4	26.3	26.1	26.3	25.1
0.375	25.2	27.1	27.2	27.4	27.6	28.4	25.1
0.435	25.2	29.1	29.4	29.4	29.6	30.2	24.7
0.485	25.3	30.1	30.3	30.4	30.5	30.7	25.2
0.55	25.4	31.2	31.4	31.5	31.6	31.8	24.7
0.625	25.3	32.4	32.5	32.6	32.7	32.5	25.2

**Table 4:** Image ‘Barb’ PSNR vs. bitrate comparison.

Bpp	PSNR in Decibels						
	**LBG**	**PSO**	**QPSO**	**HBMO**	**FA**	**BA**	**FLBG**
0.15	23.7	24.2	23.6	23.4	23.4	24.2	23.2
0.25	23.7	24.1	24.1	24.1	24.1	24.5	23.2
0.325	24.1	25.7	25.7	25.7	26.2	26.2	23.7
0.375	24.1	27.1	27.2	27.1	27.2	27.5	24.2
0.435	24.1	28.1	28.3	28.2	28.4	28.3	23.8
0.485	24.7	28.7	28.7	28.8	29.1	29.1	24.5
0.55	24.5	30.1	30.2	29.7	29.8	30.2	24.1
0.625	25.1	30.2	30.1	29.7	29.8	30.8	24.8

**Table 5:** Image ‘Goldhill’ PSNR vs. bitrate comparison.

Bpp	PSNR in Decibels						
	**LBG**	**PSO**	**QPSO**	**HBMO**	**FA**	**BA**	**FLBG**
0.15	24.4	24.5	24.2	24.2	24.2	24.8	23.6
0.25	25.1	25.2	25.2	25.5	25.7	25.8	24.7
0.325	25.5	25.2	25.8	25.8	26.1	26.1	25.1
0.375	25.6	26.1	26.1	26.8	26.7	27.1	25.1
0.435	25.6	27.2	27.3	27.6	27.7	28.2	25.2
0.485	25.6	28.2	28.1	28.7	28.6	28.8	24.8
0.55	25.6	29.7	29.8	30.1	30.1	30.2	24.7
0.625	25.6	30.1	30.1	30.4	30.3	30.8	24.8

**Table 6:** The average computational times taken across different test/experimental images.

Size of Codebook: 16								
**Image**	**Average Time Taken for Computation (seconds) at Bitrate = 0.25**							
	LBG	PSO-LBG	QPSO-LBG	HBMO-LBG	FA-LBG	BA-LBG	CS-LBG	FLBG
**CAMERAMAN**	8.13	591.11	618.56	1232.22	1173.37	599.61	2521.45	3.12
**PEPPER**	8.92	487.57	493.45	1105.28	1040.34	630.44	3326.92	3.33
**BABOON**	9.44	669.84	695.21	1983.12	1964.46	698.98	3031.06	4.13
**GOLDHILL**	9.64	625.37	740.91	1158.50	1130.75	513.28	2480.95	4.66
**BARB**	9.21	555.67	656.91	1567.51	1549.53	690.42	2811.53	4.87
**Average**	9.07	585.91	641.01	1409.33	1371.69	626.55	2834.38	4.02
**Percentage Improvement**	55.65	99.31	99.37	99.71	99.71	99.36	99.86	
**Size of Codebook: 32**								
**Image**	**Average Time Taken for Computation (seconds) at Bitrate = 0.3125**							
	LBG	PSO-LBG	QPSO-LBG	HBMO-LBG	FA-LBG	BA-LBG	CS-LBG	FLBG
**CAMERAMAN**	9.16	521.32	554.42	1291.34	1298.46	593.81	2209.83	4.12
**PEPPER**	9.82	532.17	428.92	898.76	934.76	546.91	1713.63	4.59
**BABOON**	8.88	468.12	497.96	1249.01	1243.71	549.34	2715.31	3.93
**GOLDHILL**	7.72	476.64	538.46	1340.21	1299.82	480.45	2625.02	3.06
**BARB**	10.03	423.93	474.92	1349.01	1320.15	422.78	2025.72	5.23
**Average**	9.12	484.44	498.94	1225.67	1219.38	518.66	2257.90	4.19
**Percentage Improvement**	54.11	99.14	99.16	99.66	99.66	99.19	99.81	
**Size of Codebook: 64**								
**Image**	**Average Time Taken for Computation (seconds) at Bitrate = 0.3750**							
	LBG	PSO-LBG	QPSO-LBG	HBMO-LBG	FA-LBG	BA-LBG	CS-LBG	FLBG
**CAMERAMAN**	11.31	665.23	685.12	1563.76	1491.62	671.45	2982.76	5.41
**PEPPER**	11.31	597.42	599.24	1247.54	1278.45	636.77	4468.23	5.63
**BABOON**	12.25	573.61	590.12	1412.32	1437.11	740.02	3984.18	6.12
**GOLDHILL**	14.31	622.21	637.74	1577.14	1181.33	498.56	4305.03	7.11
**BARB**	16.73	460.21	466.24	1306.21	854.17	398.74	2721.12	8.32
**Average**	13.18	583.74	595.69	1421.39	1248.54	589.11	3692.26	6.52
**Percentage Improvement**	50.55	98.88	98.91	99.54	99.48	98.89	99.82	
**Size of Codebook: 128**								
**Image**	**Average Time Taken for Computation (seconds) at Bitrate = 0.4375**							
	LBG	PSO-LBG	QPSO-LBG	HBMO-LBG	FA-LBG	BA-LBG	CS-LBG	FLBG
**CAMERAMAN**	16.28	645.34	657.54	1080.44	1054.19	628.82	1932.31	8.94
**PEPPER**	19.41	600.32	675.32	1132.68	1081.51	623.62	2220.82	10.71
**BABOON**	22.61	467.57	536.07	1112.14	1060.77	963.91	2785.66	11.11
**GOLDHILL**	18.21	835.27	860.38	1413.08	1343.73	502.21	1962.02	9.92
**BARB**	30.34	579.21	589.54	1291.81	1271.32	562.24	2438.01	12.12
**Average**	21.37	625.54	663.77	1206.03	1162.30	656.16	2267.76	10.56
**Percentage Improvement**	50.58	98.31	98.41	99.12	99.09	98.39	99.53	
**Size of Codebook: 256**								
**Image**	**Average Time Taken for Computation (seconds) at Bitrate = 0.50**							
	LBG	PSO-LBG	QPSO-LBG	HBMO-LBG	FA-LBG	BA-LBG	CS-LBG	FLBG
**CAMERAMAN**	23.15	898.23	922.46	824.23	816.44	696.46	1627.23	13.24
**PEPPER**	18.33	760.12	760.12	1010.65	984.54	574.41	1750.66	13.98
**BABOON**	28.25	599.51	568.24	1019.86	1040.91	572.32	2039.75	14.13
**GOLDHILL**	29.92	931.61	560.64	850.06	834.32	981.93	2978.06	14.33
**BARB**	27.84	689.71	698.66	847.23	837.72	596.06	2598.43	13.82
**Average**	25.50	775.84	702.02	910.41	902.79	684.24	2198.83	13.90
**Percentage Improvement**	45.49	98.21	98.02	98.47	98.46	97.97	99.37	
**Size of Codebook: 512**								
**Image**	**Average Time Taken for Computation (seconds) at Bitrate = 0.5625**							
	LBG	PSO-LBG	QPSO-LBG	HBMO-LBG	FA-LBG	BA-LBG	CS-LBG	FLBG
**CAMERAMAN**	36.84	731.32	758.23	1125.23	1078.35	849.45	1643.64	19.13
**PEPPER**	39.31	934.72	887.53	665.21	650.81	533.07	1371.76	19.12
**BABOON**	20.71	657.02	715.02	712.05	723.12	803.57	1158.42	16.32
**GOLDHILL**	35.14	582.97	601.74	955.58	960.07	885.02	1253.42	18.33
**BARB**	72.52	815.84	706.91	878.68	872.67	693.68	1805.61	36.23
**Average**	40.90	744.37	733.89	867.35	857.00	752.96	1446.57	21.83
**Percentage Improvement**	46.64	97.07	97.03	97.48	97.45	97.10	98.49	
**Size of Codebook: 1024**								
**Image**	**Average Time Taken for Computation (seconds) at Bitrate = 0.625**							
	LBG	PSO-LBG	QPSO-LBG	HBMO-LBG	FA-LBG	BA-LBG	CS-LBG	FLBG
**CAMERAMAN**	66.74	1532.32	1572.27	1918.71	2013.43	1576.42	3679.84	35.46
**PEPPER**	63.44	1022.78	1156.53	1254.02	1231.37	855.74	2059.35	32.13
**BABOON**	66.55	1435.45	1439.54	1664.23	1636.13	1665.13	2396.33	34.13
**GOLDHILL**	94.67	1353.84	1369.65	1489.71	1483.25	775.30	2340.92	48.36
**BARB**	112.32	1515.02	1503.81	1199.27	1181.88	1133.72	2400.28	58.23
**Average**	80.74	1371.88	1408.36	1505.19	1509.21	1201.26	2575.34	41.66
**Percentage Improvement**	48.40	96.96	97.04	97.23	97.24	96.53	98.38	

## Data Availability

Data will be made available upon request to M.B.
